# Comparison of Corneal Power and Astigmatism between Simulated Keratometry, True Net Power, and Total Corneal Refractive Power before and after SMILE Surgery

**DOI:** 10.1155/2017/9659481

**Published:** 2017-03-22

**Authors:** Yishan Qian, Yan Liu, Xingtao Zhou, Rajeev Krishnan Naidu

**Affiliations:** ^1^Department of Ophthalmology, Eye and ENT Hospital, Fudan University and Key Laboratory of Myopia of State Health Ministry, Shanghai, China; ^2^School of Medicine, The University of Sydney, Camperdown, NSW 2006, Australia

## Abstract

*Purpose*. To compare the mean corneal power (Km) and total astigmatism (Ka) estimated by three methods: simulated keratometry (simK), true net power (TNP), and total corneal refractive power (TCRP) before and after femtosecond laser small incision lenticule extraction (SMILE) surgery. *Methods*. A retrospective, cross-sectional study. SimK, TNP, and TCRP from a Scheimpflug analyzer were obtained from 144 patients before and 6 months after SMILE surgery. Km and Ka were recorded as the mean of individual paracentral rings of 1.0 to 8.0 mm (R1 to R8). The surgically induced changes in Km (delta-simK, delta-TNP, and delta-TCRP) and Ka (delta-simKa, delta-TNPa, and delta-TCRPa) were compared to the changes in spherical equivalent of the cycloplegic refraction (delta-SE) and astigmatism (delta-RA). *Results*. Preoperatively, astigmatism values were greatest with simKa from R1 to R5 and greatest with TCRPa from R6 to R8. Astigmatism values were smallest with TNPa from R1 to R7. Postoperatively, astigmatism values were greatest with simKa from R1 to R5 and greatest with TCRPa from R6 to R8. Delta-TCRP_3_ and Delta-TCRP_4_ matched delta-SE most closely, and delta-TCRPa_3_ matched delta-RA most closely. *Conclusions*. TCRP proved to be the most accurate method in estimating corneal power and astigmatism both before and after SMILE surgery.

## 1. Introduction

With advancements in technology, the ability to measure corneal power and other corneal parameters is becoming more and more precise. With an increase in the number of cases of refractive surgery occurring each year, the need for accurate and reliable measures of corneal power will continue to become more apparent. Although several measures currently exist, their lack of congruity and agreement has led to the need to evaluate which measures are available to consistently provide the most accurate estimates of corneal power and astigmatism. The measurements of total corneal power and astigmatism by corneal tomography are influenced by several factors: inclusion of one or two corneal surfaces, refractive index, refractive effect (e.g., the spherical aberration), and the location of the principal planes [1, 2].

Conventional keratometry (simulated keratometry) measures the paracentral anterior surface of the cornea (usually 3 mm in diameter) and calculates corneal power using a standard keratometric index of 1.3375. The standard keratometric index is based on the assumption that the corneal thickness is constant and the ratio between the curvatures of the anterior and posterior surfaces is constant. True net power (TNP) is the corneal power calculated using Gaussian optics. TNP measures both the anterior and posterior surfaces of the cornea and uses the real refractive index values of air, cornea, and aqueous. It is assumed to be more accurate than simulated keratometry (simK). However, the Gaussian optics formula is a simplified formula that uses a paraxial approximation and assumes that the rays propagating through the posterior surface of the cornea are parallel. Following laser ablation of the cornea, the above assumptions are no longer valid due to a change in both the corneal thickness and the ratio of the anterior/posterior radius of curvature, leading to an error in the measurement of corneal power and astigmatism [1–5]. Moreover, neither simK nor TNP takes the refractive effect into consideration and could be inaccurate in measuring peripheral corneal power.

Total corneal refractive power (TCRP) is calculated using Snell's law of refraction by exact ray tracing. All of the four factors mentioned above are taken into account. It is claimed to be more accurate than TNP and simK, especially in eyes following myopic laser ablation. Several studies comparing the accuracy of simK, TNP, and TCRP in the measurement of corneal power have been published [6–11]. However, no study has been conducted in the evaluation of corneal astigmatism. Moreover, past studies have primarily evaluated the central corneal power and the profile of the peripheral cornea has not yet been thoroughly investigated.

The purpose of this study was to investigate the corneal power and astigmatism distribution measured by three methods: simK, TNP, and TCRP, both before and after femtosecond laser small incision lenticule extraction (SMILE) surgery. To evaluate the accuracy of the three methods in the measurement of surgically induced changes in corneal power and astigmatism, a comparison was made between them and the changes in cycloplegic refraction following refractive surgery.

## 2. Methods

### 2.1. Patients

144 patients who underwent surgery for the correction of myopia and myopic astigmatism by femtosecond laser small incision lenticule extraction surgery were recruited between March 2013 and December 2013 to participate in this retrospective study. The study was conducted at the Ophthalmology Department of the Eye and ENT Hospital, Shanghai, China. The inclusion criteria consisted of subjects who had spherical refractions between −1.00 and −10.00 diopters (D) and astigmatism between −0.25 and −5.00 D. Additionally, best-corrected distance visual acuity values of 20/25 or better were required along with a stable refraction over two years prior to surgery, an absence of other pathologic ocular conditions or relevant systemic diseases, and a minimum of 6 months' follow-up. This study followed the tenets of the Declaration of Helsinki and was approved by the ethics committee of the EENT Hospital of Fudan University. Informed written consent was obtained from all subjects prior to participating in the study.

### 2.2. Surgical Technique

All surgeries were performed by the same surgeon (ZX). The femtosecond laser small incision lenticule extraction procedures were performed using a VisuMax femtosecond laser system (Carl Zeiss Meditec AG, Jena, Germany) following the surgical procedure outlined by Sekundo et al. [12]. The intended thickness of the upper tissue arcade (the cap) was 120 *μ*m with an intended diameter of 7.5 mm. The diameter of the refractive lenticule was 6.2 mm to 6.5 mm. A single side cut of 90 degrees with a circumferential length of 2 mm was made in the superior position. No intraoperative or postoperative complications were observed.

### 2.3. Assessment of Refractive Changes and Corneal Power

The patients were followed 1 day, 1 week, 1 month, 3 months, and 6 months after surgery. The preoperative and 6 months' postoperative measurement results were analyzed. Objective and subjective refraction assessments were performed before and after cycloplegia. The values were adjusted for the corneal plane using a vertex distance of 12 mm. Additionally, uncorrected and corrected distance visual acuities (UDVA and CDVA) were recorded.

Pentacam (Oculus GmbH, Wetzlar, Germany) imaging of the cornea was performed by the same experienced examiner, and three measurements were averaged for each result. Only the scans marked “OK” by the instrument were saved and analyzed. Corneal power was measured and shown in the power distribution display for the three methods: simK, TNP, and TCRP. simK was derived from the axial curvature map and was calculated with the keratometric index (*n* = 1.3375). TNP is the corneal power calculated using the Gaussian formula as follows:

(1) $$\begin{array}{c} F1+F2-\left(\frac{d}{n}\right)\left(F1\times F2\right), \end{array}$$

where *F*1 is the anterior corneal power, *F*2 is the posterior corneal power, *d* is the pachymetry result, and *n* = 1.376, which is the refractive index of the cornea. In the Pentacam program, TNP was calculated as the sum of *F*1 and *F*2. TCRP was calculated as the corneal power using the ray-tracing method. Corneal thicknesses and curvatures of the anterior and posterior surfaces were obtained by Scheimpflug imaging. The calculation of TCRP is based on Snell's law of refraction using real refractive index values (1 for air, 1.376 for the cornea, and 1.336 for aqueous). Corneal power is then determined by

(2) $$\begin{array}{c} \frac{n}{f}, \end{array}$$

where *n* = 1.336 and *f* is the focal length, referenced to the anterior corneal surface. Values (*K*_steep_, *K*_flat_, and axis of *K*_flat_) demonstrated by individual rings centered on the pupil center ranging from 1.0 mm to 8.0 mm (R1 to R8) and the mean values of the central 3 mm zone were recorded. The arithmetic mean of the steep and flat corneal axes and the difference between them (the astigmatism) were calculated and compared. The surgically induced changes in corneal power (delta-simK, delta-TNP, and delta-TCRP) were compared to the changes in cycloplegic refraction of spherical equivalent (delta-SE) which were calculated by subtracting the postoperative SE from the preoperative SE.

Vector analysis was performed to decompose the cylinder notation into two cross cylinder components, *J*_0_ and *J*_45_ [13, 14]. *J*_0_ and *J*_45_ are defined as

(3) $$\begin{array}{c} J_{0}=-\left(\frac{C}{2}\right)cos\left(2\alpha \right),\\ J_{45}=-\left(\frac{C}{2}\right)sin\left(2\alpha \right), \end{array}$$

where *C* is the magnitude of the cylinder power present and *α* is the axis in radians. *J*_0_ represents the horizontal/vertical component of astigmatism and *J*_45_ represents astigmatism with axes at 45° and 135°. Vector mean (power ∗ axis) is calculated as

(4) $$\begin{array}{c} power=2\sqrt{{J_{0}^{2}}_{mean}+{J_{45}^{2}}_{mean}},\\ axis=0.5\times arctan\left(\frac{J_{45mean}}{J_{0mean}}\right). \end{array}$$

The following two indices were used to compare corneal astigmatisms: the magnitude of the vector difference between two corneal astigmatisms and the absolute value of the angular difference between two corneal astigmatisms which was defined always to be an acute angle.

The surgically induced astigmatism (SIA) is the amount and axis of astigmatic change caused by surgery. It was determined as the vector difference between the pre- and postoperative refractive astigmatism (or corneal astigmatism) [13, 14]. The surgically induced changes in corneal astigmatism (delta-simKa, delta-TNPa, and delta-TCRPa) were compared to the changes in cycloplegic refraction of astigmatism using Bland-Altman analysis [15].

### 2.4. Statistical Analysis

Statistical analysis was performed using a commercially available statistical software package (SPSS, ver. 13.0; SPSS, Chicago, IL). Comparisons between the three methods were performed using analysis of variance (ANOVA) and least significant difference (LSD) analyses. Comparisons between refractive changes and corneal power changes were performed using a paired *t*-test. A *P* value of less than 0.05 was considered statistically significant. The bias between delta-refractive astigmatism (delta-RA) and delta-corneal astigmatism (delta-Ka) was evaluated using 95% limits of agreement. 95% limits of agreement were the mean difference ± 1.96 × standard deviation of the difference [15].

## 3. Results

The right eyes of 144 patients (88 females (61.1%)) were included in the analyses. The mean age of patients was 26.3 ± 6.6 years, with a range of 18 to 41 years. The mean preoperative and postoperative cycloplegic spherical errors at the corneal plane were −5.73 ± 1.33 D (range: −1.71 to −8.12 D) and 0.12 ± 0.51 D (range: −1.95 to +1.53 D), respectively. The mean preoperative and postoperative cycloplegic astigmatisms at the corneal plane were −0.92 ± 0.61 D (range: −3.70 to 0.00 D) and −0.10 ± 0.21 D (range: −1.22 to 0.00 D), respectively. The mean preoperative CDVA (logarithm of minimal angle of resolution (logMAR)) was −0.02 ± 0.06 (range: −0.18 to 0.30). The mean postoperative UDVA and CDVA were −0.02 ± 0.08 (range: −0.18 to 0.40) and −0.05 ± 0.06 (range: −0.20 to 0.10), respectively. Delta-spherical equivalent (delta-SE) was 6.61 ± 1.37 D, and delta-refractive astigmatism (delta-RA) determined by vector analysis was (0.58 ± 0.61 D) × 90.8°, respectively.

### 3.1. Preoperatively

**Figure 1:**
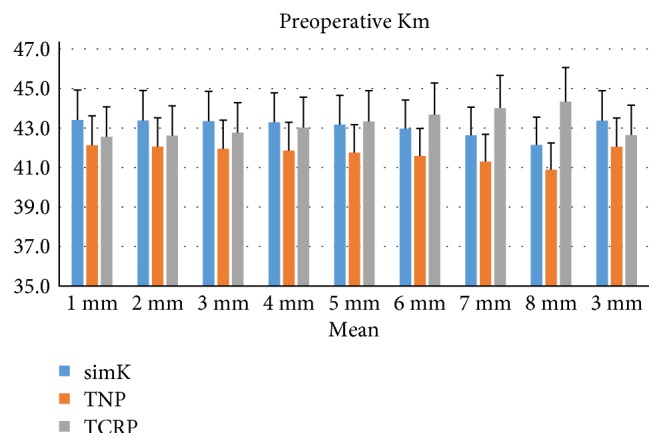
Preoperative corneal power of R1 to R8 and mean of the central 3 mm zone estimated by the three methods: simulated keratometry (simK), true net power (TNP), and total corneal refractive power (TCRP). Significant differences were found between the three corneal powers from R1 to R8 and the central 3 mm (ANOVA: *P* < 0.05).

Significant differences were found between the three corneal powers from R1 to R8 (ANOVA: *P* < 0.05, Figure 1). TNP produced the smallest estimates among the three corneal powers from R1 to R8 (LSD: *P* < 0.05). simKs were greater than TCRPs from R1 to R3, and TCRPs were greater than simKs from R6 to R8 (LSD: *P* < 0.05). When calculating the mean of the central 3 mm zone, simK estimates (43.31 ± 1.46 D) were the greatest and TNP estimates (41.92 ± 1.40 D) were the smallest among the three corneal powers (LSD: *P* < 0.001).

The vector means of three corneal astigmatisms and the comparisons between them are listed in Table 1. Astigmatism values were greatest with simKa from R1 to R5 and greatest with TCRPa from R6 to R8. Astigmatism values were smallest with TNPa from R1 to R7. The absolute values of angular difference between TCRPa and simKa were greatest with R1 and smallest with R4; the absolute values of angular difference between TCRPa and TNPa were greatest with R8 and smallest with R3 to R5; the absolute values of angular difference between simKa and TNPa were greatest with R1 and smallest with R4 and R5.

### 3.2. Postoperatively

**Figure 2:**
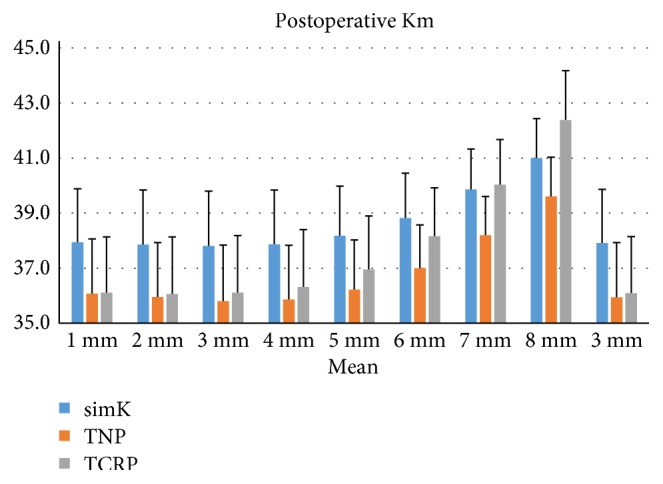
Postoperative corneal power of R1 to R8 and mean of the central 3 mm zone estimated by three methods: simulated keratometry (simK), true net power (TNP), and total corneal refractive power (TCRP). Significant differences were found between the three corneal powers from R1 to R8 and the central 3 mm (ANOVA: *P* < 0.05).

Postoperatively, Kms of simK, TNP, and TCRP were all significantly decreased from the preoperative Kms for R1 to R8 (paired *t*-test, *P* < 0.05). simKs were greater than TNPs for R1 to R8 (LSD: *P* < 0.05, Figure 2). simKs were greater than TCRPs for R1 to R6 and smaller than TCRP for R8 (LSD: *P* < 0.05). TCRPs were greater than TNPs for R4 to R8 (LSD: *P* < 0.05). The mean postoperative corneal power of the central 3 mm zone was greatest with simK (37.90 ± 1.96 D) and smallest with TNP (35.95 ± 2.00 D, LSD: *P* < 0.05).

The vector means of postoperative corneal astigmatisms are listed in Table 2. When compared to the preoperative Ka, astigmatism values were decreased for R1 to R5 and increased for R6 to R8, as measured by all the three methods. Astigmatism values were greatest with simKa from R1 to R5 and greatest with TCRPa from R6 to R8. TNPa and TCRPa matched each other well from R1 to R5. The absolute values of angular difference in astigmatism between TCRPa and simKa were greatest with R2 and smallest with R6; the absolute values of angular difference in astigmatism between TCRPa and TNPa were greatest with R8 and smallest with R3 to R5; the absolute values of angular difference between simKa and TNPa were greatest with R2 and smallest with R6.

### 3.3. Surgically Induced Changes in Corneal Power (Delta-Km) and Astigmatism (Delta-Ka)

**Figure 3:**
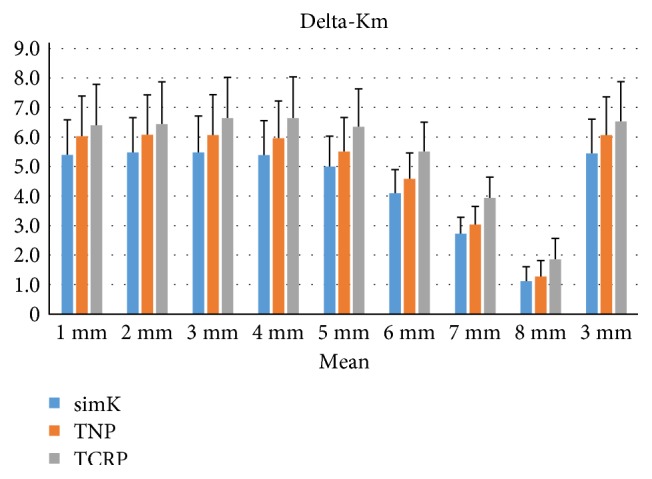
Delta-corneal power of R1 to R8 and mean of the central 3 mm zone estimated by three methods: simulated keratometry (simK), true net power (TNP), and total corneal refractive power (TCRP). Significant differences were found between the three corneal powers from R1 to R8 and the central 3 mm (ANOVA: *P* < 0.05).

Delta-Kms were greatest with delta-TCRP and smallest with delta-simK from R1 to R8 (LSD: *P* < 0.05, Figure 3). Delta-TCRP for R3 (6.63 ± 1.20 D) and R4 (6.64 ± 1.16 D) matched delta-SE (6.61 ± 1.37 D) most closely. The SIAs calculated by vector analyses, the means of differences between the vector values of delta-RAs and delta-Kas, the standard deviations, and the 95% limits of agreement are listed in Table 3. Delta-RA (0.58 × 90.8°) was smaller than delta-Ka of R1 and R2 measured by all the three methods. Delta-TCRPa of R3 (0.57 × 94.8°) matched delta-RA most closely. Delta-TNPa of R3 (0.52 × 94.4°) and delta-simKa of R3 (0.46 × 94.7) underestimated delta-RA.

## 4. Discussion

The need for an accurate and reliable measure of corneal power and astigmatism is becoming more apparent, especially in the constantly advancing setting of corneal refractive surgery. While several methods already exist to measure corneal power and astigmatism, many of them lack great consistency and accuracy between them. Some of the commonly used methods also make assumptions or use parameters in their estimations that are not valid following refractive surgery. The aim of this study was to evaluate the accuracy of three commonly used measures of corneal power and astigmatism before and after myopic corneal refractive surgery. The accuracy of these three measures was determined through comparisons between their estimated surgically induced changes in corneal power and astigmatism with the changes in cycloplegic refraction obtained clinically following refractive surgery.

In the current study, total corneal power was calculated by three methods: simK, TNP, and TCRP. In the central cornea prior to corneal refractive surgery, simK estimated the greatest values of corneal power while TNP estimated the smallest of the three measures. This has been confirmed in several studies in which TCRP was found to be most accurate, while simK overestimated and TNP underestimated the central corneal power [2–4]. This could be a result of the keratometric index error involved in the simK calculation and the paraxial approximation used in the TNP calculation. Moreover, the different reference planes used by the different methods may also contribute to the error. The ray-tracing method uses the anterior corneal surface as the reference plane, whereas the Gaussian optics formula uses the second principal plane immediately in the front of the cornea.

When analyzing the peripheral cornea, we found that TCRPs increased from the central to peripheral cornea, while both simK and TNP decreased from the central to peripheral cornea. The distribution of TCRPs reflected the positive spherical aberration of the cornea [16]. Consequently, TCRP should be adopted when an accurate measurement of peripheral corneal power is needed, as it most closely parallels the changes in the higher-order aberration across the corneal profile from the center to the periphery. Although TCRP has been claimed to be the most accurate measure of corneal power, there is currently a very limited number of studies investigating its application. Therefore, this approach needs to be validated and optimized before it can be adopted for routine use in the measurement of corneal power. Moreover, since the current intraocular lens (IOL) formulas are based on keratometry values, TCRP might not be utilized in these formulas until a calculation based on the ray-tracing method has been established.

The results of the current study obtained following refractive surgery were consistent with those of previous studies [3–11], demonstrating that simK significantly overestimated postoperative central corneal power while TNP significantly underestimated postoperative central corneal power. Although TCRPs slightly overestimated [6, 9] or underestimated [8] delta-SE, they matched delta-SE more closely than delta-simK or delta-TNP. The errors induced by the assumptions used in the calculations of simK and TNP were exaggerated by corneal ablation, which changed both the corneal thickness and the ratio of the anterior/posterior radius of curvature. We also found that delta-TCRP of the 3.0 mm and 4.0 mm rings matched delta-SE most closely. Savini et al. [6] found that the delta-TCRP of the 2.0 mm ring and the delta-TCRP of the central 3.0 mm zone provided the closest approximation to the delta-SE after myopic refractive surgery. Savini et al. [7] also demonstrated that TCRP calculated over the entrance pupil ensured the most accurate estimation of delta-SE. Oh et al. [8] established that the delta-TCRP of the 4 mm zone exhibited the least difference with the noncycloplegic delta-SE following myopic refractive surgery. The different results obtained in the studies mentioned above could be related to the variable sizes of the optical zone, the variable ablation profiles, and the different methods of calculation (pupil or apex center, ring or zone) used in the individual investigations. Therefore, the conclusion of an individual investigation should not be generalized across all procedures and should be applied cautiously to other laser surgical platforms.

The accuracy of TCRP in the measurement of astigmatism in virginal cornea has been proved in several studies where TCRP has been used as the gold standard [17–19]. We found that simK produced the largest and TNP produced the smallest estimates of the three corneal astigmatism measurements in the central cornea. We also found that TCRP and TNP matched each other much better than simK, demonstrating that the error involved in the power calculation of simK also exists in the astigmatic calculation. Roh et al. [20] compared astigmatism measurements using automated keratometry versus TCRP on cataract patients and found that TCRPs were more accurate in astigmatism measurements in highly irregular corneas. Calossi [16] found that the difference in astigmatism between simK and TCRP depends on the axis orientation. simK overestimates TCRP (mean 0.11 ± 0.22 D) in the eyes with with-the-rule astigmatism, and simK underestimates TCRP (mean 0.26 ± 0.31 D) in the eyes with against-the-rule astigmatism. Consequently, the eyes of with-the-rule and against-the-rule (as well as oblique) astigmatisms should be evaluated separately. Unfortunately, since the majority of the eyes in the current study had with-the-rule astigmatism and the sample sizes were very small for the other two types of astigmatism, any comparison between them is difficult. Further study with more eyes with against-the-rule and oblique astigmatisms will be done to clarify this question.

To our knowledge, this is the first study evaluating the profile of total corneal astigmatism as a function of corneal diameters. We found that astigmatism measured by simK increased from 1 mm to 4 mm and then decreased. No significant difference was found for TNP from 1 mm (1.18 D) to 8 mm (1.34 D). Astigmatism measured by TCRP increased from 1 mm (1.04 D) to 8 mm (1.50 D). An increase in corneal astigmatism from the center to the periphery has also been reported by Mathur et al. [21]. Consequently, TCRP should be used in the measurement of peripheral corneal astigmatism, for example, in the evaluation of contact lens fittings, since the peripheral corneal contour could influence the fitting success of contact lenses [22, 23].

As for the postoperative corneal astigmatism, we found that delta-Ka of R1 and R2 measured by all the three methods overestimated delta-RA. Delta-TCRPa of R3 matched delta-RA most closely while both delta-TNPa_3_ and delta-simKa_3_ underestimated delta-RA. Thus, TCRP proved to be accurate in the estimates of both corneal power and corneal astigmatism after refractive surgery. The current results may provide significant contributions to contact lens fitting following refractive surgery, as they can provide practitioners with a tool to evaluate changes in the central and peripheral corneal astigmatisms as a function of postoperative manifest refractive changes [24, 25].

We also found that all postoperative Kms (from R1 to R8) were decreased from the preoperative TCRPs. This is beyond the size of the lenticule, as well as the size of the cap. In contrast, the postoperative astigmatism values were decreased from the preoperative Ka from R1 to R5 and increased from R6 to R8. The creation of the cap and the postoperative changes in peripheral corneal contour after the removal of the lenticule may contribute to the peripheral changes in corneal power [26]. Additional studies applying other imaging systems, such as anterior segment optical coherence tomography (AS-OCT), could possibly contribute to solving this question. The changes in corneal power beyond the size of the lenticule can create a transitional zone-like area, which may help improve the visual quality.

A limit of this study was that the higher order aberrations (HOAs) were not included in the analyses. Several studies have reported significant increases in HOAs following SMILE surgery, especially increases in the coma. The change in the corneal asphericity, as well as the change in pupil diameter, may have an effect on the postoperative corneal astigmatism [27–29]. Further investigations, including pre- and postoperative HOAs, would help to clarify this problem.

In conclusion, simK overestimated the central corneal power (and astigmatism) and TNP underestimated the central corneal power (and astigmatism) both before and after refractive surgery. TCRP proved to be the most accurate method among them, especially after refractive surgery. The distribution of TCRPs reflected the profile of positive spherical aberration across the cornea. Delta-TCRP of R3 and R4 matched the cycloplegic delta-SE most closely. Delta-TCRPa of R3 matched delta-RA most closely. Although the determination of TCRP has been claimed to be the most accurate measure of corneal power, this approach needs to be further validated and optimized before it can be adopted for routine use in the measurement of corneal power and astigmatism in a clinical setting.

## Figures and Tables

**Table 1:** Comparisons of the preoperative corneal cylindrical errors between simulated keratometry (simKa), true net power (TNPa), and total corneal refractive power (TCRPa) from 1 mm to 8 mm. All values were adjusted for the corneal plane using a vertex distance of 12 mm.

Vector means				Difference					
	simKa	TNPa	TCRPa	TCRPa versus simKa		TCRPa versus TNPa		simKa versus TNPa	
				Magnitude^∗^	|Angle|^∗∗^	Magnitude^∗^	|Angle|^∗∗^	Magnitude^∗^	|Angle|^∗∗^
1 mm	(0.94 ± 0.56) × 91.2	(0.71 ± 0.57) × 89.7	(0.74 ± 0.57) × 89.9	0.20 ± 0.20	6.9 ± 12.2	0.03 ± 0.13	1.1 ± 4.7	0.23 ± 0.15	6.9 ± 12.2
2 mm	(1.11 ± 0.57) × 91.3	(0.88 ± 0.57) × 90.3	(0.93 ± 0.58) × 90.6	0.18 ± 0.16	4.6 ± 9.4	0.05 ± 0.18	1.5 ± 8.2	0.24 ± 0.15	5.0 ± 11.0
3 mm	(1.17 ± 0.55) × 91.6	(0.97 ± 0.54) × 91.7	(1.02 ± 0.55) × 91.9	0.14 ± 0.14	3.1 ± 6.0	0.05 ± 0.13	0.8 ± 5.1	0.20 ± 0.09	2.8 ± 4.4
4 mm	(1.19 ± 0.56) × 92.4	(1.02 ± 0.53) × 93.1	(1.11 ± 0.57) × 93.1	0.09 ± 0.13	2.9 ± 5.5	0.08 ± 0.10	0.8 ± 5.0	0.17 ± 0.09	2.6 ± 4.0
5 mm	(1.15 ± 0.57) × 94	(1.01 ± 0.57) × 94.6	(1.13 ± 0.60) × 94.9	0.04 ± 0.14	3.1 ± 5.8	0.13 ± 0.19	0.8 ± 4.1	0.14 ± 0.15	2.6 ± 4.7
6 mm	(1.05 ± 0.59) × 96.3	(0.98 ± 0.57) × 96.6	(1.12 ± 0.65) × 97.1	0.07 ± 0.16	3.3 ± 7.3	0.14 ± 0.14	1.2 ± 3.3	0.07 ± 0.11	2.7 ± 6.4
7 mm	(0.95 ± 0.60) × 98.2	(0.95 ± 0.58) × 97.2	(1.12 ± 0.69) × 97.9	0.16 ± 0.36	3.7 ± 8.2	0.17 ± 0.17	1.6 ± 4.1	0.03 ± 0.10	3.5 ± 7.2
8 mm	(0.91 ± 0.61) × 98.2	(1.01 ± 0.61) × 95.7	(1.25 ± 0.77) × 96.5	0.53 ± 0.98	5.3 ± 10.9	0.25 ± 0.26	2.5 ± 9.1	0.13 ± 0.11	4.4 ± 8.0

^∗^Magnitude represented the magnitude of the vector difference.

^∗∗^|Angle| was defined as the mean absolute values of the arithmetic difference between the axis of two corneal astigmatisms. It was defined always to be an acute angle.

**Table 2:** Comparisons of the postoperative corneal cylindrical errors among simulated keratometry (simKa), true net power (TNPa), and total corneal refractive power (TCRPa) from 1 mm to 8 mm. All values were adjusted for the corneal plane using a vertex distance of 12 mm.

Vector means				Difference					
	simKa	TNPa	TCRPa	TCRPa versus simKa		TCRPa versus TNPa		simKa versus TNPa	
				Magnitude^∗^	|Angle|^∗∗^	Magnitude^∗^	|Angle|^∗∗^	Magnitude^∗^	|Angle|^∗∗^
1 mm	(0.25 ± 0.62) × 75.2	(0.21 ± 0.70) × 36.9	(0.20 ± 0.69) × 39.2	0.27 ± 0.19	10.5 ± 13.2	0.02 ± 0.09	1.3 ± 6.1	0.29 ± 0.18	9.9 ± 11.6
2 mm	(0.49 ± 0.56) × 83.8	(0.24 ± 0.61) × 69.7	(0.24 ± 0.61) × 69.2	0.30 ± 0.15	12.0 ± 14.9	0.01 ± 0.08	1.3 ± 8.2	0.30 ± 0.15	11.2 ± 12.7
3 mm	(0.71 ± 0.47) × 89.6	(0.46 ± 0.48) × 88.6	(0.46 ± 0.49) × 88.3	0.25 ± 0.12	11.4 ± 15.7	0.01 ± 0.05	0.6 ± 0.9	0.25 ± 0.12	11.2 ± 15.5
4 mm	(0.92 ± 0.47) × 92.3	(0.74 ± 0.49) × 93.2	(0.75 ± 0.50) × 93.4	0.18 ± 0.12	6.1 ± 10.4	0.01 ± 0.04	0.6 ± 0.9	0.19 ± 0.12	5.8 ± 9.8
5 mm	(1.07 ± 0.52) × 94.0	(0.93 ± 0.51) × 94.6	(0.98 ± 0.54) × 94.9	0.10 ± 0.13	3.5 ± 5.9	0.05 ± 0.05	0.6 ± 1.0	0.14 ± 0.12	3.2 ± 5.2
6 mm	(1.16 ± 0.68) × 95.8	(1.07 ± 0.67) × 95.7	(1.17 ± 0.74) × 96.0	0.01 ± 0.14	3.3 ± 4.8	0.10 ± 0.08	0.8 ± 1.3	0.09 ± 0.11	2.7 ± 3.9
7 mm	(1.15 ± 0.84) × 97.5	(1.14 ± 0.85) × 96.5	(1.31 ± 1.00) × 96.7	0.17 ± 0.25	4.5 ± 7.2	0.17 ± 0.17	1.1 ± 1.8	0.04 ± 0.15	3.7 ± 6.5
8 mm	(1.09 ± 0.93) × 98.2	(1.19 ± 0.98) × 96.4	(1.44 ± 1.25) × 97.6	0.35 ± 0.47	5.8 ± 11.3	0.26 ± 0.39	3.7 ± 10.9	0.12 ± 0.15	2.9 ± 4.1

^∗^Magnitude represented the magnitude of the vector difference.

^∗∗^|Angle| was defined as the mean absolute values of the arithmetic difference between the axis of two corneal astigmatisms. It was defined always to be an acute angle.

**Table 3:** Surgically induced changes in astigmatisms (SIA) calculated by vector analyses in delta-refractive astigmatisms (delta-RAs) and delta-corneal astigmatisms (delta-Kas), means of differences between the vectors of delta-RAs and delta-Kas, and 95% limits of agreement (LOA).

	SIA (D × degree)	*J*_0_ [(delta-RA)–(delta-Ka)]		*J*_45_ [(delta-RA)–(delta-Ka)]	
		Mean	95% LOA^∗^	Mean	95% LOA^∗^
Delta-RA	(0.58 ± 0.61 D) × 90.8	na	na	na	na
Delta-simKa					
1 mm	(0.74 ± 0.70) × 96.4	0.07	−0.63 to 0.77	0.07	−0.58 to 0.72
2 mm	(0.65 ± 0.64) × 96.8	**0.03**	−0.51 to 0.56	0.07	−0.44 to 0.58
3 mm	(0.46 ± 0.58) × 94.7	−0.06	−0.53 to 0.41	0.03	−0.34 to 0.40
4 mm	(0.27 ± 0.55) × 92.8	−0.16	−0.70 to 0.39	0.005	−0.37 to 0.38
5 mm	(0.08 ± 0.43) × 94.2	−0.25	−0.94 to 0.44	−**0.003**	−0.42 to 0.42
6 mm	(0.11 ± 0.40) × 0.42	−0.34	−1.32 to 0.63	−0.01	−0.58 to 0.56
7 mm	(0.19 ± 0.53) × 3.6	−0.39	−1.63 to 0.86	−0.02	−0.73 to 0.69
8 mm	(0.18 ± 0.68) × 8.1	−0.38	−1.74 to 0.98	−0.03	−0.83 to 0.76
Delta-TNPa					
1 mm	(0.80 ± 0.80) × 97.1	0.10	−0.67 to 0.86	0.09	−0.63 to 0.81
2 mm	(0.72 ± 0.71) × 96.8	0.06	−0.53 to 0.65	0.08	−0.50 to 0.65
3 mm	(0.52 ± 0.64) × 94.4	−**0.04**	−0.50 to 0.43	0.03	−0.36 to 0.43
4 mm	(0.29 ± 0.60) × 92.7	−0.15	−0.70 to 0.40	0.005	−0.39 to 0.40
5 mm	(0.08 ± 0.50) × 94.7	−0.25	−1.00 to 0.49	−**0.002**	−0.43 to 0.42
6 mm	(0.09 ± 0.42) × 176.7	−0.33	−1.32 to 0.65	−0.003	−0.59 to 0.58
7 mm	(0.19 ± 0.57) × 2.7	−0.38	−1.67 to 0.90	−0.02	−0.75 to 0.72
8 mm	(0.18 ± 0.76) × 10.5	−0.37	−1.80 to 1.06	−0.04	−0.89 to 0.81
Delta-TCRPa					
1 mm	(0.80 ± 0.79) × 97.0	0.10	−0.65 to 0.85	0.09	−0.63 to 0.80
2 mm	(0.78 ± 0.74) × 96.6	0.09	−0.49 to 0.66	0.08	−0.48 to 0.64
3 mm	(0.57 ± 0.65) × 94.8	−**0.008**	−0.46 to 0.45	0.04	−0.35 to 0.43
4 mm	(0.36 ± 0.63) × 92.6	−0.11	−0.66 to 0.44	0.008	−0.38 to 0.40
5 mm	(0.16 ± 0.50) × 94.9	−0.21	−0.90 to 0.47	**0.005**	−0.40 to 0.41
6 mm	(0.06 ± 0.45) × 166.0	−0.32	−1.32 to 0.69	0.006	−0.56 to 0.58
7 mm	(0.20 ± 0.64) × 179.7	−0.39	−1.76 to 0.98	−0.007	−0.78 to 0.76
8 mm	(0.19 ± 0.99) × 14.9	−0.37	−2.08 to 1.34	−0.06	−1.05 to 0.94

^∗^95% limits of agreement = mean difference ± 1.96 × standard deviation of the difference.

simK: simulated keratometry; TNP: true net power; TCRP: total corneal refractive power.
